# B-cell translocation gene 2 enhances fibroblast growth factor 21 production by inducing Kruppel-like factor 15

**DOI:** 10.1038/s41598-019-40359-2

**Published:** 2019-03-06

**Authors:** Yong Deuk Kim, Seung-Lark Hwang, Hwang-Ju Jeon, Yong Hyun Jeon, Balachandar Nedumaran, Kyeongsoon Kim, Sung-Eun Lee

**Affiliations:** 1 School of Applied Biosciences, Kyungpook National University, Daegu, 41566 Republic of Korea; 2 Laboratory Animal Center, Daegu-Gyeongbuk Medical Innovation Foundation, Daegu, 41061 Republic of Korea; 3 Division of Cardiothoracic Surgery, Department of Surgery, School of Medicine, University of Colorado Anschutz Medical Campus, Aurora, Colorado USA; 4 Department of Pharmaceutical Engineering, Inje University, Gimhae, 50834 Republic of Korea

## Abstract

Fibroblast growth factor 21 (FGF21) is a hormone that is vital for the regulation of metabolic homeostasis. In the present study, we report that Kruppel-like factor 15 (KLF15) is a novel mediator of b-cell translocation gene 2 (BTG2)-induced FGF21 biosynthesis. The expression levels of hepatic *Fgf21*, *Btg2*, and *Klf15*, and the production of serum FGF21 increased significantly in fasted and forskolin (FSK)-treated mice. The overexpression of *Btg2* using an adenoviral delivery system elevated FGF21 production by upregulating *Klf15* transcription. Interaction studies indicated that BTG2 was co-immunoprecipitated with KLF15 and recruited by the *Fgf21* promoter. The disruption of hepatic *Btg2* and *Klf15* genes markedly attenuated the induction of *Fgf21* expression and FGF21 biosynthesis in fasted mice. Similarly, the FSK-mediated induction of *Fgf21* promoter activity was strikingly ablated by silencing of *Btg2* and *Klf15*. Taken together, these findings suggest that KLF15 and BTG2 are mediators of fasting-induced hepatic FGF21 expression. Therefore, targeting BTG2 and KLF15 might be a therapeutically important strategy for combat metabolic dysfunction.

## Introduction

Fibroblast growth factor 21 (FGF21) is a member of the FGF superfamily and acts as an endocrine hormone. It is produced at high levels in several tissues, including the adipocytes, liver, muscle, and heart^1^. The concentration of hepatic FGF21 increased under various pathophysiological conditions, including fasting, starvation, ketogenic diet, fatty liver disease, obesity, and mitochondrial dysfunction^2,3^. FGF21 plays a key role in diverse physiological functions, such as gluconeogenesis, ketogenesis, steatosis, and inflammation^1,4^. Moreover, FGF21 enhances insulin sensitivity and fatty acid oxidation and attenuates fat accumulation, pro-inflammatory cytokines, reactive oxygen species, and growth hormone resistance by controlling several transcription factors, including peroxisome proliferator-activated receptor α (PPARα), farnesoid X receptor (FXR), activating transcription factor 4 (ATF4), and nuclear factor-kappa B (NF-κB)^1,5,6^.

B-cell translocation gene 2 (BTG2) belongs to the BTG/Tob protein family, which is characterized by the presence of two homology domains (Box A and B) that are highly conserved among diverse species^7^. BTGs are anti-proliferative factors and are key modulators of cellular manifestations, such as cell growth, proliferation, differentiation, death, and survival^7,8^. It is mainly expressed in the liver and has also been detected in diverse tissues, including the kidney, skeletal muscle, lung, pancreas, and intestine^9^. BTG2 levels are increased by hypoxia, oxidative stress, metabolic changes, and retinoic acid, whereas they are reduced by insulin, estrogen, and growth factors^10^. Interestingly, we demonstrated previously that BTG2, a transcriptional co-regulator, enhances the expression of several target genes, including insulin and hepcidin^10,11^.

Kruppel-like factor 15 (KLF15), also known as kidney-enriched KLF (KKLF), is a member of the KLF family of transcription factors. Member of this family contain a zinc finger DNA-binding domain that is highly conserved among diverse species. KLF15 is predominantly expressed in the liver, but there is also significant expression in other tissues, including the kidney, pancreas, muscle, and heart^12^. KLF15 expression is upregulated in diverse tissues by glucagon and glucocorticoids during starvation or under diabetic conditions, whereas feeding and insulin downregulate KLF15 expression^12,13^. KLF15 also regulates the transcription of target genes involved in diverse physiological processes, including fibrosis, cardiovascular disease, and the immune response. It is associated with metabolic dysfunction, including cardiac hypertrophy, obesity, inflammation, and diabetes^12,14^. Gluconeogenic signals are known to modulate FGF21 production via the activation of glucocorticoid receptor (GR) and PPARα in mice subjected to prolonged fasting or starvation^15,16^. We have previously shown that BTG2 is induced under glucagon and fasting condition^7,11^. Moreover, KLF15 is known to be upregulated by glucagon and downregulated by insulin^12,13^. However, the connection of BTG2 and KLF15 in the regulation of FGF21 production is largely unknown.

In the current study, we demonstrate that BTG2 is a key modulator of FGF21 production and reveal a novel molecular mechanism that links KLF15 to the control of FGF21 biosynthesis during starvation.

## Results

### Gluconeogenic signals elevate FGF21 gene expression and biosynthesis

**Figure 1:**
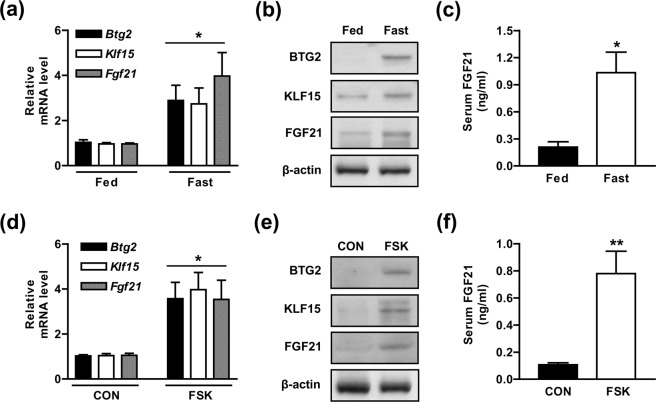
Fasting state and forskolin exposure elevate hepatic FGF21 gene expression and production in the liver. (**a)** Wild-type **(**WT) mice were fed *ad libitum* and fasted for 24 h. Gene expression levels were measured by qPCR analysis with various primers. (**b)** Tissue extracts were analyzed by Western blot analysis using specific antibodies. (**c)** Serum FGF21 levels at the indicated conditions. (**d)** WT mice were injected intraperitoneally with forskolin (FSK, 5 mg/kg body weight) for 6 h, and then assessed by qPCR analysis with gene-specific primers. (**e)** Tissue extracts were analyzed by Western blot analysis with various antibodies. (**f)** Serum FGF21 levels in the indicated groups of mice; n = 7 mice per group. *P < 0.05, **P < 0.01 *vs*. fed mice or untreated control mice.

Previous studies have showed that gluconeogenic signals modulate FGF21 production via the GR and PPARα in mice during prolonged fasting and/or starvation^15,16^. Based on these findings, we examined the physiological connection between gluconeogenic modulators and hepatic FGF21 metabolism. Notably, the expression of *Fgf21* was significantly elevated under prolonged fasting, along with the induction of *Btg2* and *Klf1*5 expression when compared with that of the fed state (Fig. 1a). Consistent with the increase in *Fgf21*, *Btg2*, and *Klf15* mRNA expression, the corresponding protein levels were markedly elevated in the livers of the fasted mice (Fig. 1b). In addition, serum FGF21 concentrations was also increased in the fasted mice compared to the fed state (Fig. 1c). Furthermore, we examined the critical effect of gluconeogenic signaling on the regulation of *Fgf21* expression and the secretion of FGF21 in the liver. FSK treatment enhanced the protein and mRNA levels of FGF21, BTG2, and KLF15 (Fig. 1d,e). Likewise, FSK challenge also increased the serum FGF21 concentration relative to that of the control groups (Fig. 1f). Collectively, these findings suggested a potential link between BTG2 and FGF21 biosynthesis in response to gluconeogenic signals.

### BTG2 elevates FGF21 production via the induction of KLF15

**Figure 2:**
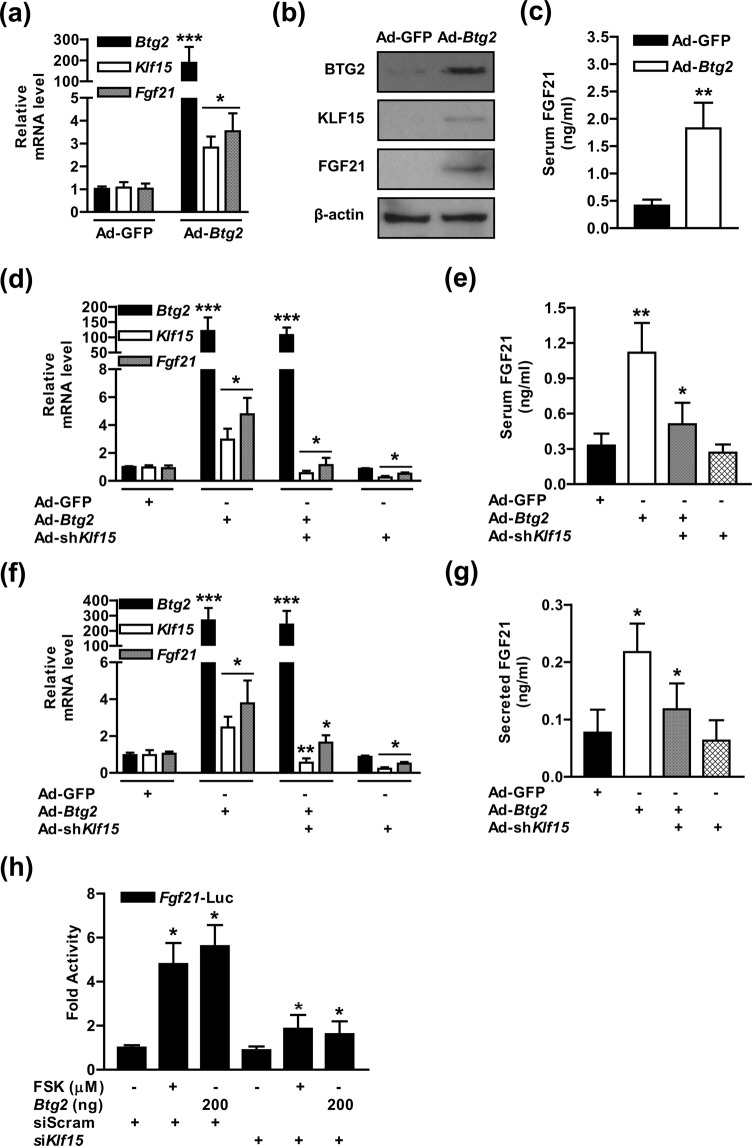
BTG2 increases FGF21 gene expression and biosynthesis. (**a)** WT mice were tail-vein injected with Ad-GFP and Ad-*Btg2* for 7 days. qPCR analysis showing *Btg2*, *Klf15*, *Fgf21* expression in the liver. (**b)** Tissue extracts were analyzed by Western blot analysis with the indicated antibodies. (**c)** Serum FGF21 production in the observed mice. **(d)** WT mice were intravenously injected with Ad-GFP, Ad-*Btg2*, and Ad-sh*Klf15* for 7 days. Total RNAs were isolated from the mouse livers, and the expression levels of the various genes were determined by qPCR analysis with specific primers. **(e)** Serum FGF21 production in the indicated mice; n = 7 mice per group. **(f)** AML12 cells were infected with Ad-GFP, Ad-*Btg2*, and Ad-sh*Klf15* for 36 h, and then analyzed by qPCR with various primers. **(g)** The culture media in the AML12 cells was harvested for FGF21 secretion analysis as indicated. (**h)** AML12 cells were transiently transfected with si*Klf15* and siScram. After transfection for 36 h, the cells were co-transfected with the indicated reporter gene and *Btg2* and subjected to FSK treatment for 6 h. *P < 0.05, **P < 0.01, ***P < 0.001 *vs*. Ad-GFP, Ad-*Btg2*, untreated controls, or FSK-treated cells.

We have attempted to investigate the critical role of BTG2 as a key modulator of FGF21 biosynthesis using an adenoviral delivery system expressing *Btg2* (Ad-*Btg2*) or a control green fluorescent protein (Ad-GFP) in mouse livers. Ad-*Btg2* was successfully delivered to the livers of wild-type (WT) mice via tail vein injection. The expression levels of *Klf15* and *Fgf21* were significantly higher in the Ad-*Btg2*-infected mice than in the Ad-GFP control mice (Fig. 2a,b). As expected, Ad-*Btg2* significantly increased the serum FGF21 level compared to that in the Ad-GFP control mice (Fig. 2c). Next, to determine whether BTG2-mediated induction of FGF21 expression and production can be modulated by KLF15, we assessed the effect of *Klf15* on the regulation of FGF21 gene expression and biosynthesis using adenoviral-mediated overexpression of *Btg2* (Ad-*Btg2*) and knockdown of *Klf15* (Ad-sh*Klf15*) both *in vivo* and *in vitro*. Ad-*Btg2* effectively enhanced *Klf15* and *Fgf21* mRNA levels, while this phenomenon was markedly negated by silencing of *Klf15* in mouse livers and hepatocytes (Fig. 2d,f). Similarly, the production of FGF21 increased by Ad-*Btg2* was remarkably decreased in *Klf15* knockdown hepatocytes and mouse livers (Fig. 2e,g). Interestingly, Ad-sh*Klf15* slightly reduced basal *Fgf21* expression in the hepatocytes and mice relative to the Ad-GFP control group, but not the production of FGF21 (Fig. 2d–g). To further confirm the transcriptional activity of *Fgf21* by FSK treatment, *Btg2*, *Klf15*, we investigated *Fgf2*1 promoter activity in hepatocytes. Notably, FSK exposure or transiently expressed *Btg2* significantly elevated the promoter activity of *Fgf21*, whereas this stimulation was strikingly blunted in *Klf15* knockdown groups compared to the control groups (Fig. 2h). Overall, these results demonstrate that BTG2 acts as a major modulator of FGF21 gene expression and biosynthesis by depending on KLF15 both *in vivo* and *in vitro*.

### Gluconeogenic signal-stimulated FGF21 production depends on BTG2

**Figure 3:**
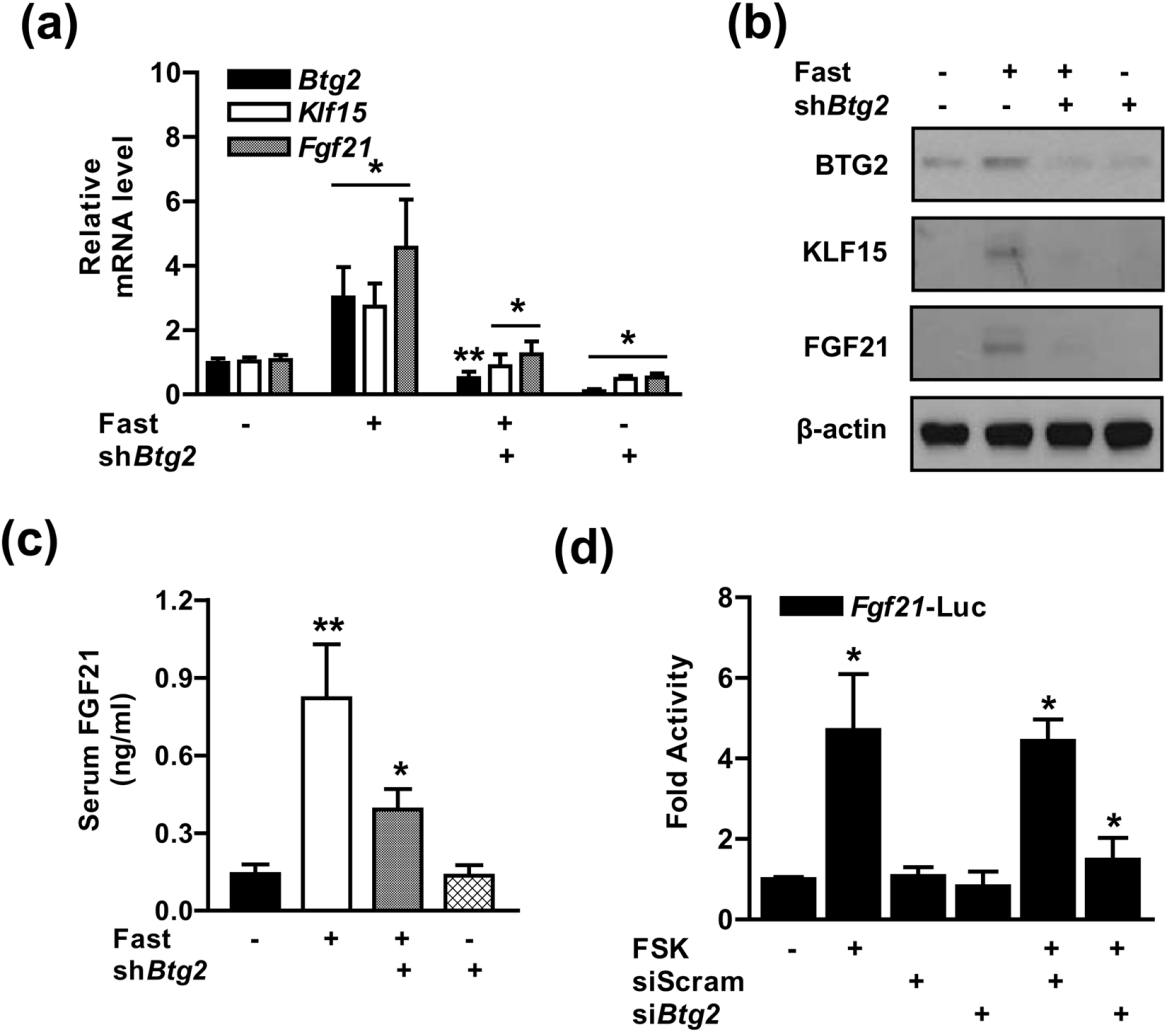
Elevation of FGF21 production by fasting and forskolin treatment is mediated by BTG2. (**a)** WT mice were tail-vein injected with lentivirus-sh*Btg2* (sh*Btg2*). After 7 days, the mice were fasted for 24 h. Total RNAs were isolated from the mouse livers and expression levels were determined by qPCR analysis with various primers. (**b)** Western blot analysis showing BTG2, KLF15, and FGF21 expression in the mouse livers. (**c)** Serum levels of FGF21 in the indicated group of mice; n = 7 mice per group. (**d)** AML12 cells were transfected with si*Btg2* and siScram. After transfection for 36 h, the cells were transiently transfected with the indicated reporter genes and subjected to FSK treatment for 6 h. *P < 0.05, **P < 0.01 *vs*. untreated controls, fasted mice, or forskolin-treated cells.

We further investigated the pivotal role of BTG2 in fasting-mediated FGF21 metabolism using lentiviral-mediated knockdown of *Btg2* (sh*Btg2*) in mouse livers. The expression of *Btg2* was successfully attenuated in the mouse livers. The elevation of *Btg2*, *Klf15*, and *Fgf21* mRNA and protein levels during fasting were markedly alleviated by endogenous *Btg2* knockdown (Fig. 3a,b). As anticipated, the increase of serum FGF21 concentration induced by fasting was strikingly reduced in the *Btg2* knockdown mice (Fig. 3c). Moreover, basal FGF21 expression was weakly attenuated in *Btg2*-silenced mice compared to the control group, but not the secretion of FGF21 (Fig. 3a–c). Next, we further verified whether BTG2 modulates the transcriptional activity of *Fgf21* in hepatocytes. As shown in Fig. 3d, *Fgf21* promoter activity was enhanced by FSK exposure, and this stimulatory effect of FSK was markedly diminished when *Btg2* was silenced. Taken together, these findings suggest that BTG2 plays an important role in modulating FGF21 production during fasting.

### KLF15 is required for fasting-induced FGF21 expression and biosynthesis

**Figure 4:**
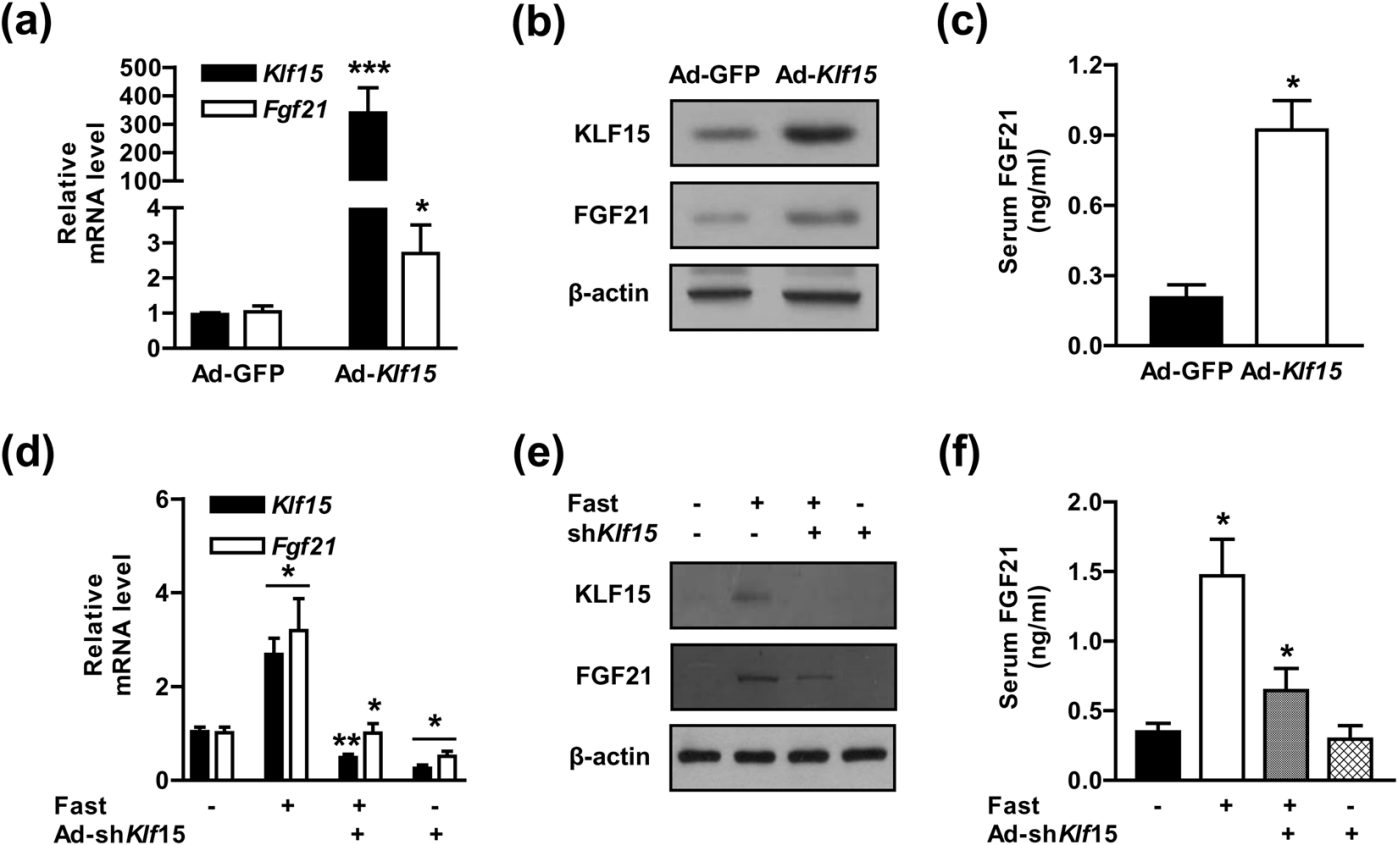
KLF15 regulates fasting-induced FGF21 expression and biosynthesis. (**a)** WT mice were tail-vein injected with Ad-GFP and Ad-*Klf15* for 7 days. Total RNA expression levels were measured by qPCR analysis with gene-specific primers. (**b)** Tissue extracts were analyzed by Western blot analysis with various antibodies. (**c)** Serum levels of FGF21 in the observed mice. (**d)** WT mice were tail-vein injected with Ad-sh*Klf15* for 7 days. The mice were then fasted for 24 h. Gene expression levels were analyzed using qPCR with various primers. (**e)** Western blot analysis showing KLF15 and FGF21 expression in the mouse livers. (**f)** Levels of serum FGF21 in the indicated mice; n = 7 mice per group. *P < 0.05, **P < 0.01, ***P < 0.001 *vs*. Ad-GFP or fasted mice.

We explored the possible effects of KLF15 on FGF21 gene regulation and production in response to gluconeogenic stimuli in mouse livers using Ad-*Klf15*. As shown in Fig. 4a,b, qPCR and Western blot analysis showed the successful overexpression of *Klf15* in mouse livers. Ad-*Klf15* significantly elevated *Fgf21* gene expression (Fig. 4a,b), and consequently increased serum FGF21 levels compared with those in the control groups (Fig. 4c). We further investigated the direct effect of KLF15 on fasting-induced FGF21 gene expression and biosynthesis using Ad-sh*Klf15*. The expression of *Klf15* in the liver of mice was successfully decreased by Ad-sh*Klf15* delivery. The increase in *Fgf21* gene expression induced by fasting was markedly attenuated by *Klf15* silencing (Fig. 4d,e). Moreover, the fasting-induced serum FGF21 concentration was dramatically diminished by the knockdown of endogenous *Klf15* (Fig. 4f). Notably, basal *Fgf21* expression was slightly reduced in the *Klf15*-silenced mice relative to the control group, but not the production of FGF21 (Fig. 4d–f). Collectively, these observations strongly suggest that gluconeogenic stimuli guide FGF21 gene expression and production and are partially dependent on KLF15.

### BTG2 physically interacts with KLF15 and controls KLF15 occupancy on the *Fgf21* promoter

**Figure 5:**
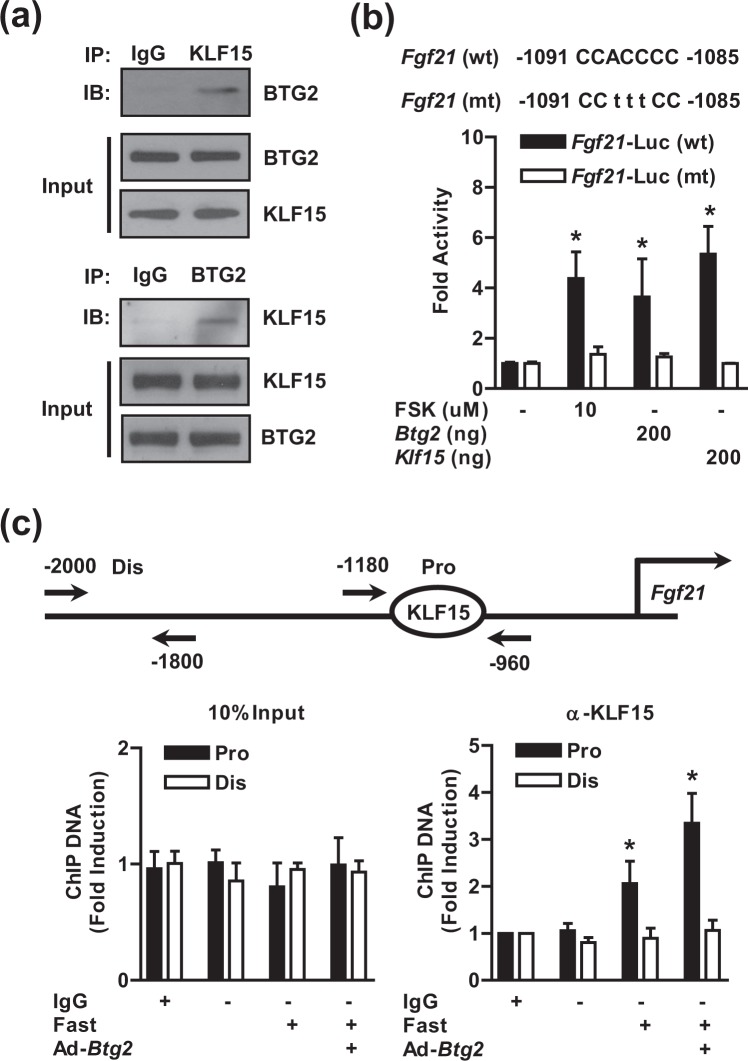
BTG2 associates with KLF15 and is recruited on the *Fgf21* promoter. (**a)** WT mice were fed *ad libitum* and fasted for 24 h. Co-immunoprecipitation (Co-IP) assays with liver extracts indicated the association between BTG2 and KLF15. Protein extracted from the livers was immunoprecipitated with either KLF15 or BTG2 antibodies, and then immunoblotted with the same antibodies. (**b)** Schematic diagrams of the KLF15 binding site on the WT *Fgf21* promoter construct from −1091 to −1085 bp and its mutant form (mt). AML12 cells were transfected with the indicated reporter genes by *Btg2* or *Klf15* co-transfection and subjected to FSK treatment for 6 h. (**c)** Chromatin immunoprecipitation (ChIP) assay showing the occupancy of KLF15 on the *Fgf21* promoter in the liver. WT mice were infected with Ad-*Btg2* for 7 days and fasted for 24 h. Input was the 10% of soluble chromatin. The remaining soluble chromatin was subjected to immunoprecipitation using anti-KLF15 antibodies or IgG as indicated. Purified DNA samples were utilized for qPCR analysis with specific primers binding to the indicated regions (proximal and distal region) on the *Fgf21* promoter. *P < 0.05 *vs*. untreated controls or fasting mice.

To further elucidate whether BTG2 and KLF15 in the regulation of *Fgf2*1 gene transcription via physically interaction, we carried out co-immunoprecipitation (Co-IP) assays using lysates from fasted and fed mouse livers. Endogenous BTG2 interacted strongly with KLF15 protein in the fasted mice compared to the fed mice (Fig. 5a). We identified the KLF15-binding site on the *Fgf21* promoter using *in silico* analysis. Our reporter gene assay indicated that *Btg2* and *Klf15* alone increased *Fgf21* promoter activity, whereas this stimulation was completely negated in the KLF15-binding site-mutated *Fgf21* promoter compared to the control group (Fig. 5b). To further confirm that BTG2 affects the recruitment of KLF15 protein to the *Fgf21* promoter, we carried out using a chromatin immunoprecipitation (ChIP) assay with an anti-KLF15 antibody in the mouse livers. As expected, endogenous KLF15 occupancy of the proximal (Pro) region was significantly enhanced by fasting and/or Ad-*Btg2* compared to that in the control groups. However, this effect was totally lost in the nonspecific distal (Dis) region of the *Fgf21* promoter (Fig. 5c). Overall, these results strongly suggest that BTG2 associates with KLF15 and enhances *Fgf21* transcription by recruiting KLF15.

## Discussion

In the current study, we demonstrated that fasting and/or FSK treatment remarkably induced hepatic FGF21 gene transcription, and BTG2 elevated FGF21 biosynthesis via the upregulation of KLF15 expression. Conversely, the stimulatory effect of fasting or FSK exposure on hepatic FGF21 gene expression and production was prominently attenuated by the disruption of *Btg2* and *Klf15* expression in mouse livers and hepatocytes. Therefore, we propose that the fasting-BTG2-KLF15 signaling network may represent a novel molecular mechanism underlying the modulation of hepatic FGF21 gene expression and its biosynthesis.

Several previous reports have shown that gluconeogenic signals modulate hepatic FGF21 gene expression and biosynthesis in rodents^15–17^. Our previous study revealed that the elevation of BTG2 by glucagon increased hepcidin levels and gluconeogenesis in mouse livers^11,18^. However, the potent effect of BTG2 in modulating hepatic FGF21 gene expression and FGF21 biosynthesis remains largely unexplored. The results of the current study demonstrate that enhanced BTG2 levels induced by fasting or FSK challenge modulate hepatic FGF21 production by upregulating the expression of *Klf15*. Fasting and FSK treatment significantly increase the expression of *Btg2*, *Klf15*, and *Fgf21* in mouse livers and significantly elevate the concentration of serum FGF21 (Fig. 1). Ad-*Btg2* significantly increased hepatic FGF21 gene expression and production by stimulating KLF15 expression (Fig. 2), whereas this stimulation was markedly attenuated in *Btg2* knockdown cells and mice (Fig. 3). Our findings suggest that BTG2 plays a crucial role in modulating the fasting-mediated induction of hepatic FGF21 gene expression and biosynthesis via the induction of KLF15.

KLF15 modulates the transcription of several target genes that are involved in various physiological processes, such as fibrosis, cardiac hypertrophy, obesity, inflammation, insulin resistance, and diabetes^12–14,19,20^. Based on these findings, we investigated the molecular mechanism underlying fasting-stimulated *Fgf21* gene transcription by the BTG2-KLF15 pathway both *in vivo* and *in vitro*. First, the increase in FGF21 gene expression induced by fasting was markedly diminished by silencing of *Klf15* relative to that in the control groups (Fig. 4). Second, BTG2 strongly interacted with KLF15 in the liver lysates from fasted mice compared to the lysates from fed mice. Third, the endogenous KLF15 occupancy of the proximal region of the *Fgf21* promoter was effectively enhanced by fasting and Ad-*Btg2* transduction; and the transcriptional activity of *Fgf21* was significantly increased by *Btg2* and *Klf15* in the hepatocytes (Fig. 5). Collectively, our results reveal a connection between *Fgf21* transcription and the BTG2-KLF15 axis. However, we cannot rule out the possibility that the detailed molecular mechanism that connects FGF21 gene expression and the BTG2-KLF15 signaling network depends on unknown mechanisms such as an association with transcriptional co-activators and competition with co-repressors, microRNAs, and protein stability to modulate hepatic FGF21 gene transcription.

**Figure 6:**
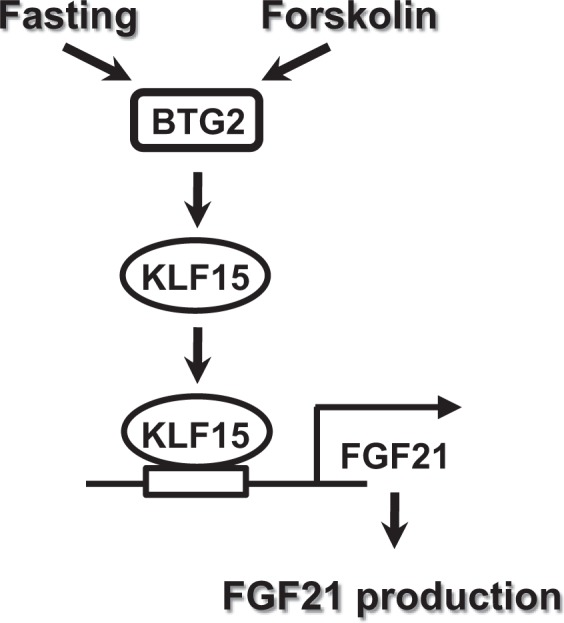
Schematic model illustrating the regulation of FGF21 biosynthesis by the BTG2-KLF15 signaling network. Fasting and forskolin treatment significantly induce the expression of FGF21 by enhancing the BTG2-KLF15 signaling network, which subsequently increases FGF21 production.

In conclusion, the present study demonstrate that FGF21 is a novel target of BTG2, and that BTG2 promotes FGF21 biosynthesis by upregulating the expression of *Klf15* during fasting or FSK exposure. These findings suggest that the elevation of BTG2 by gluconeogenic signals modulates hepatic FGF21 homeostasis by inducing the expression of *Klf15*. Therefore, as depicted in Fig. 6, a molecular mechanism involving hepatic FGF21 homeostasis in response to BTG2-KLF15 signaling may provide the basis for the development of novel therapeutic agents for the treatment of metabolic disorders.

## Materials and Methods

### Animals

We used 8-week-old male C57BL/6 mice (Samtako, Osan, Republic of Korea) for the experiments, as described below^11^. For the fasting and feeding experiments, we fed the mice *ad libitum*, and then fasted them for 24 h. For the forskolin (FSK) stimulation experiments, we injected the mice intraperitoneally with FSK (Sigma-Aldrich, St. Louis, MO, USA) at a dose rate of 5 mg/kg of body weight and left them for 6 h. For the *Btg2* and *Klf15* overexpression experiments, we injected wild-type (WT) mice with adenoviral vectors expressing *Btg2* and *Klf15* (1 × 10^9^ plaque-forming units, pfu) via their tail veins for 7 days. For the disruption of the *Btg2* and *Klf15* genes, we injected WT mice with lentivirus short hairpin RNA (shRNA) targeting *Btg2* (sh*Btg2*, a single dose of 1 × 10^9^ transducing units, TU/mL) and with Ad-sh*Klf15* (1 × 10^9^ pfu) through their tail veins for 7 days. At the end of the specified experiments or challenge periods, we euthanized the mice with CO_2_, and harvested liver tissues and blood samples. All animal experiments and protocols were approved and performed by the Institutional Animal Care and Use Committee (IACUC) of the Kyungpook National University according to the rules and guidelines of the National Institutes of Health (NIH).

### Measurement of serum FGF21

We collected the blood samples immediately, and determined serum FGF21 levels using a Quantikine FGF21 enzyme-linked immunosorbent assay (ELISA) kit (R&D Systems, Minneapolis, MN, USA) according to the manufacturer’s instructions^21^.

### Construction of plasmids and DNA

The mouse *Fgf21* promoter and *Klf15* expression vector were kind gifts from Drs. Hueng-Sik Choi (Chonnam National University, Gwangju, Republic of Korea) and Myung-Shik Lee (Yonsei University College of Medicine, Seoul, Republic of Korea), respectively^21,22^. The KLF15 response element-mutated *Fgf21* promoter was generated using a Site-Directed Mutagenesis kit (Stratagene, La Jolla, CA, USA) with the following primers: forward, 5′-CTAATCCTCCCTTTCCCCAAA-3′, and reverse, 5′-TTTGGGGAAAGGGAGGATTAG-3′. The *Btg2* expression vector has been reported previously^7^. All constructs were confirmed via sequencing analysis.

### Culture of hepatocytes and transient transfection assays

We cultured AML-12 cells (immortalized mouse hepatocytes) in Dulbecco’s modified Eagle’s medium (DMEM)/F-12 medium (Gibco-BRL, Grand Island, NY, USA) supplemented with 10% fetal bovine serum (Gibco-BRL), insulin-transferrin-selenium (Gibco-BRL), dexamethasone (40 ng/ml, Sigma-Aldrich), and antibiotics in a humidified atmosphere containing 5% CO_2_ at 37 °C, according to a previously described method^18,23^. The transient transfection assays were performed using Lipofectamine 2000 (Invitrogen, Carlsbad, CA, USA) with the indicated reporter plasmid and expression vectors encoding various genes and/or chemicals according to the manufacturer’s protocol^24^. The luciferase activity was normalized to *β*-galactosidase activity.

### Recombinant adenovirus and small interfering RNA (siRNA)

Adenoviruses encoding full-length *Btg2* (Ad-*Btg2*), green fluorescent protein (GFP), and a lentiviral delivery system for *Btg2*-targeted shRNA (sh*Btg2*) have been described previously^18^. We purchased Ad-*Klf15* and Ad-sh*Klf15* from Vector Biolabs (Malvern, PA, USA). The siRNAs for *Btg2* (siScram and si*Btg2*) and *Klf15* (siScram and si*Klf15*) were chemically manufactured (Bioneer Research, Seoul, Republic of Korea) and transfected using Oligofectamine reagent (Invitrogen) in accordance with the manufacturer’s protocol, as previously described^7^.

### Measurement of mRNA

Total RNA was extracted from each sample using the TRIzol method (Invitrogen, Carlsbad, CA, USA), as mentioned previously^25^. We synthesized complementary DNA (cDNA) using a Maxima^®^ First Strand cDNA synthesis kit (Fermentas, Vilnius, Lithuania), and used a StepOne^TM^ Real-time PCR system (Applied Biosystems, Warrington, UK) for quantitative real-time polymerase chain reaction (qPCR) analysis. We determined *Btg2*, *Klf15*, and *Fgf21* gene expression levels by qPCR, as described previously^11,19,21^. All the transcripts were normalized to ribosomal L32 expression.

### Western blot analysis

We extracted liver samples for Western blot analysis according to a previously described method^11,21^. The membranes were probed with BTG2, KLF15, and β-actin (Santa Cruz Biotechnology, Santa Cruz, CA, USA) and FGF21 (Abcam, Cambridge, UK). Immunoreactive proteins were developed using an ECL-Plus Western blot detection kit (GE Healthcare, Piscataway, NJ, USA) in accordance with the manufacturer’s protocol.

### Co-immunoprecipitation assay

We immunoprecipitated the total proteins from the liver extract with antibodies against BTG2 and KLF15 (Santa Cruz Biotechnology) using protein A/G PLUS-Agarose (Santa Cruz Biotechnology). The immunoprecipitated proteins were then subjected to Western blot analysis using the corresponding antibodies^11^. Signals were developed using an ECL-Plus Western blot detection kit (Amersham Bioscience, Piscataway, NJ, USA).

### Chromatin immunoprecipitation (ChIP) assay

The ChIP assay was carried out using the anti-KLF15 antibody, as described previously^11,18^. Briefly, soluble chromatin was subjected to immunoprecipitation using the anti-KLF15 antibody (SantaCruz Biotechnology). After recovering DNA, the purified DNA samples were utilized for qPCR with specific primers covering the (proximal and distal regions) of the *Fgf21* promoter. The primers used for PCR were as follows: proximal region, forward 5′-GGGTTCCTCCTAGAAATCCA-3′ and reverse 5′-CAGTCACCCTCACCAACCCC-3′; distal region, forward 5′-GCTGCGCCCTCTCCTCCGCC-3′ and reverse 5′-TGGGAACGT-GCATAGAACCT-3′.

### Statistics

Data and statistical analysis were performed using GraphPad Prism 3–5.0 software. The statistical significance between groups was determined by applying the Student’s *t*-test or the Mann-Whitney U-test, and multiple comparisons were analyzed using one-way analysis of variance (ANOVA) or the Kruskal Wallis test. All data are represented as the mean ± the standard error of the mean (SEM). Differences were considered statistically significant at *P*-values of <0.05.
