# Author Correction: B-cell translocation gene 2 enhances fibroblast growth factor 21 production by inducing Kruppel-like factor 15

**DOI:** 10.1038/s41598-020-59784-9

**Published:** 2020-02-13

**Authors:** Yong Deuk Kim, Seung-Lark Hwang, Hwang-Ju Jeon, Yong Hyun Jeon, Balachandar Nedumaran, Kyeongsoon Kim, Sung-Eun Lee

**Affiliations:** 1 School of Applied Biosciences, Kyungpook National University, Daegu, 41566 Republic of Korea; 2 Laboratory Animal Center, Daegu-Gyeongbuk Medical Innovation Foundation, Daegu, 41061 Republic of Korea; 3 Division of Cardiothoracic Surgery, Department of Surgery, School of Medicine, University of Colorado Anschutz Medical Campus, Aurora, Colorado USA; 4 Department of Pharmaceutical Engineering, Inje University, Gimhae, 50834 Republic of Korea; 5 Korea Bio Science Co., Daegu, 41520 Republic of Korea

Correction to: *Scientific Reports* 10.1038/s41598-019-40359-2, published online 06 March 2019

The original version of this Article omitted an affiliation for Seung-Lark Hwang. The correct affiliations for Seung-Lark Hwang are listed below:

School of Applied Biosciences, Kyungpook National University, Daegu, 41566, Republic of Korea

Korea Bio Science Co., Daegu 41520, Republic of Korea

This has now been corrected in the HTML and PDF versions of this Article.

